# An Initial Investigation of an Alternative Model to Study rat Primordial Germ Cell Epigenetic Reprogramming

**DOI:** 10.1186/s12575-017-0058-1

**Published:** 2017-08-03

**Authors:** Isabelle Hernandez Cantão, Renato Borges Tesser, Taiza Stumpp

**Affiliations:** 1 Laboratory of Developmental Biology, Department of Morphology and Genetics, Federal University of Sao Paulo (UNIFESP), Sao Paulo, Brazil; 2 Rua Botucatu, 740. Ed. Leitão da Cunha, 2o andar. CEP: 04023-900, São Paulo, Brazil

**Keywords:** Rat, Primordial germ cell, Epigenetic reprogramming

## Abstract

**Background:**

Primordial germ cells (PGC) are the precursors of the gametes. During pre-natal development, PGC undergo an epigenetic reprogramming when bulk DNA demethylation occurs and is followed by sex-specific de novo methylation. The de novo methylation and the maintenance of the methylation patterns depend on DNA methyltransferases (DNMTs). PGC reprogramming has been widely studied in mice but not in rats. We have previously shown that the rat might be an interesting model to study germ cell development. In face of the difficulties of getting enough PGC for molecular studies, the aim of this study was to propose an alternative method to study rat PGC DNA methylation. Rat embryos were collected at 14, 15 and 19 days post-coitus (dpc) for the analysis of 5mC, 5hmC, DNMT1, DNMT3a and DNMT3b expression or at 16dpc for treatment 5-Aza-CdR, a DNMT inhibitor, in vitro.

**Methods:**

Once collected, the gonads were placed in 24-well plates previously containing 45μm pore membrane and medium with or without 5-Aza-CdR. The culture was kept for five days and medium was changed daily. The gonads were either fixed or submitted to RNA extraction.

**Results:**

5mC and DNMTs labelling suggests that PGC are undergoing epigenetic reprogramming around 14/15dpc. The in vitro treatment of rat embryonic gonads with 1 μM of 5-Aza-CdR lead to a loss of 5mC labelling and to the activation of *Pax6* expression in PGC, but not in somatic cells, suggesting that 5-Aza-CdR promoted a PGC-specific global DNA hypomethylation.

**Conclusions:**

This study suggests that the protocol used here can be a potential method to study the wide DNA demethylation that takes place during PGC reprogramming.

## Background

Primordial germ cells (PGC) are peculiar cells that differentiate into gametes and transmit the genetic and epigenetic information to the next generations. In mice, PGC arise from a pool of epiblastic cells that retain the expression of pluripotent markers [1–3]. This pool of cells starts to express *fragillis* and *Blimp1*, which conduce to a germ cell fate [4–7]. PGC then migrates via the hindgut and its mesentery to reach the genital ridges, where they undergo male or female differentiation.

The switch from a somatic to a germ cell program depends initially on the maintenance of pluripotency markers and later on epigenetic reprogramming, when PGC DNA is widely hypomethylated [8–10] and then de novo hypermethylated in a sex-specific manner [11]. Mouse PGC reprogramming starts around 8dpc and ends around 16.5dpc in males, when DNA de novo methylation ends [4, 8, 10–15]. De novo DNA methylation, as well as the maintenance of methylation patterns during cell division, occur via DNA methyltransferases (DNMT). DNMT3a and DNMT3b are described as de novo DNA methyltransferases capable of establishing methylation patterns whereas DNMT1 is considered the maintenance DNMT during DNA replication.

It has been suggested that DNA demethylation in mouse PGC occurs through two mechanisms: oxidation of 5mC to 5hmC via TET enzymes and through replication-dependent dilution [16, 17]. These mechanisms operate in three steps: the first is a passive process of replicative-dilution of 5mC possibly due to a lack of DNMT1 function and occurs from 8.5dpc to 9.5dpc; the second is the active process where 5mC are oxidated to 5hmC via TET (Ten-eleven translocation) enzymes at 10.5dpc; and the third step is again a passive step that consists in the replicative-dilution of 5hmC from 10.5dpc to 13.5dpc [17].

Like in the early embryo, the de novo DNA methylation as well as the maintenance of DNA methylation also seems to be mediated by the DNMTs, although the data are controversial. DNMT1 seems to be dispensable for the reacquisition of DNA methylation in male germ cells [18, 19]. On the other hand, DNMT3a is responsible for de novo DNA methylation [18, 20], whereas DNMT3b would function in the maintenance of de novo DNA methylation [20] or would not be required in prenatal germ cell development [18].

The natural epigenetic reprogramming by which germ cells go through is one of the most interesting events of their pre-natal development. Although the meaning of this event has not been clarified, it is likely to be related to the establishment of a totipotent state that seems to be fundamental for germ cell differentiation [21] and maybe to confer the developmental potential to the future gametes. Investigations about the role of DNA methylation in gene expression have made use of agents that impair the maintenance of DNA methylation, such as 5-Aza-deoxycitidine (5-Aza-CdR), to induce artificial DNA demethylation in vitro [12, 22]. 5-Aza-CdR is known to interact with DNMT1, inhibiting its activity. This compound acts in both proliferating and non-proliferating cells [23, 24] and is able to activate genes which expression is controlled by promoter methylation [12, 22].

Although the mouse has been the main model for studies on germ cell development, we have previously shown relevant differences in the detection of germ cell markers between mice and rats [25], suggesting that the rat also seems to be an important model to study germ cell development. Despite the relatively abundant studies about PGC reprogramming, especially in the mouse, the methodologies involved are laborious due to the low number of PGC in the initial phases of their development. When the rat is the model of choice, the difficulties are increased due to the fact that less information and tools are available for this species.

In face of the technical difficulties to study the control of gene expression by DNA methylation in PGC coupled to the impossibility to establish PGC cultures that maintain their in vivo characteristics, we aimed to find new and simpler methodologies to study the mechanisms involved in DNA methylation and demethylation in rat PGC. Here we describe DNMT expression in rat embryo gonads and a method that lead to the specific hypomethylation of PGC DNA by the DNMT inhibitor 5-Aza-CdR. This study may be useful for future investigations in the field.

## Results

### Dnmt Expression and Selection of the age for 5-Aza-CdR Treatment

**Fig. 1:**
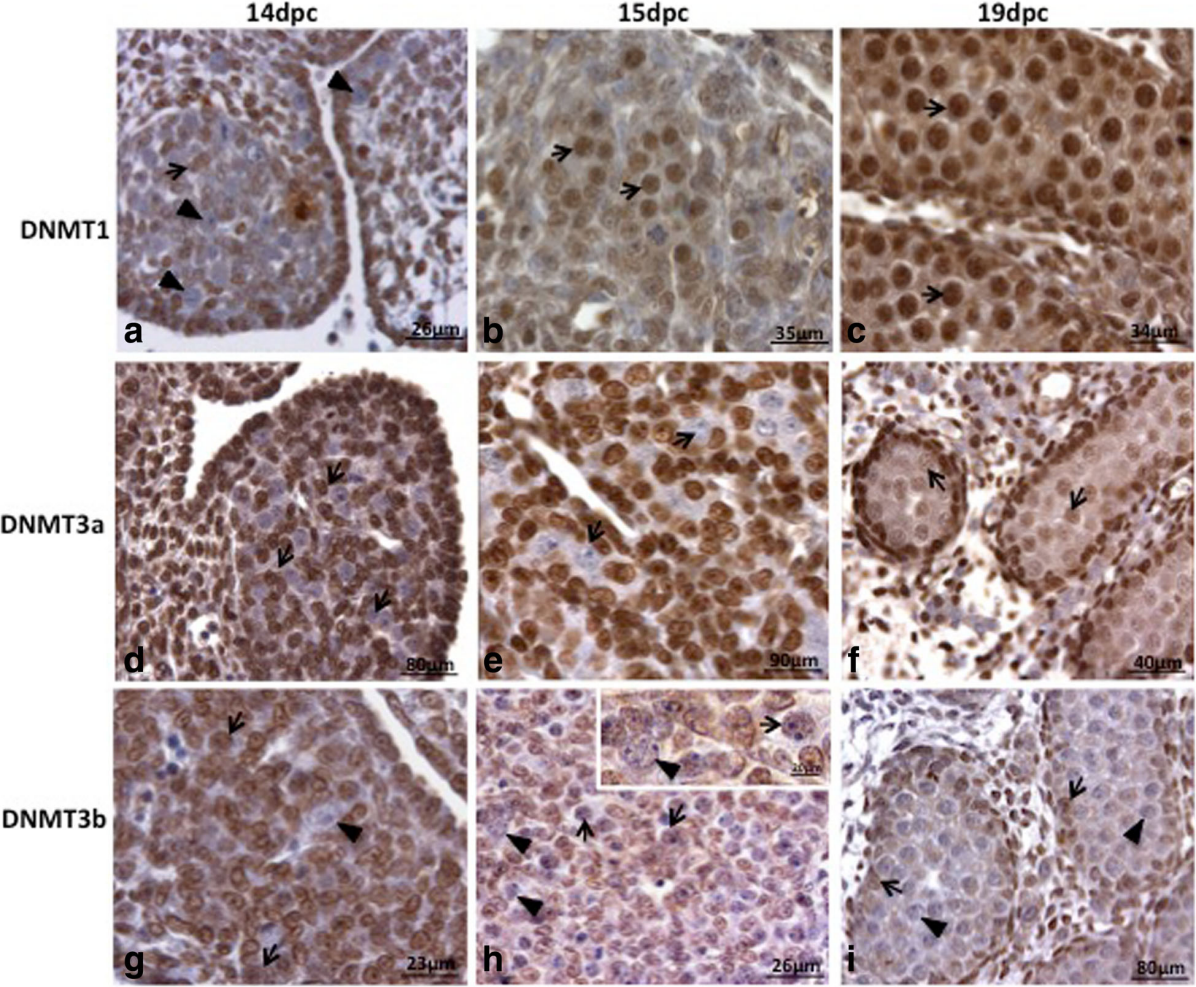
DNMT1, DNMT3a and DNMT3b labelling in male rat embryonic gonads. DNMT1 was not detected in PGC (*arrowheads*) at 14dpc (**a**); the somatic cells were positive (*arrows*). At 15dpc (**b**) and 19dpc (**c**) all PGC/gonocytes were positive (*arrows*). These results coincides with 5mC labelling, indicating that PGC reprogramming ends around 15dpc. DNMT3a was not detected in PGC (*arrows*) at 14dpc (**d**) and 15dpc (**e**). At 19dpc (**f**) DNMT3a was detected (*arrows*) in all gonocytes, although the labelling intensity has varied among them. At 14dpc (**g**) and 15dpc (**h**) DNMT3b-positive (*arrows*) and DNMT3b-negative (*arrowheads*) PGC were observed. At 19dpc (**i**) DNMT3b was not detected (*arrowheads*) in the gonocytes and was detected in Sertoli cells (*arrows*)

**Fig. 2:**
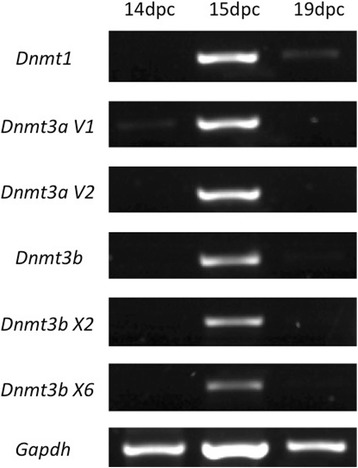
*Dnmt1*, *Dnmt3a* (variants 1 and 2) and *Dnmt3b* (variants X2 and X6) expression in rat PGC/gonocytes ate 14dpc, 15dpc and 19dpc. All the genes analysed showed similar expression pattern, with 15dpc PGC showing expression of the six mRNAs and 14dpc PGC showing no expression. An exception is observed with the expression of *Dnmt1*, which was detected at 19dpc, although the band observed was very weak

Considering that 5-Aza-CdR acts through the inhibition of DNMT1 activity [26], the presence of this enzyme was investigated in rat PGC (Figs. 1 and 2). DNMT1, which is responsible for maintaining DNA methylation (DNMT1), was not detected in PGC at 14dpc in both protein (Fig. 1a) and RNA (Fig. 2) levels, suggesting that DNMT1 activity is not necessary in PGC at this stage of rat germ cell development. On the other hand, at 15dpc (Fig. 1b) and at 19dpc (Fig. 1c) DNMT1 was detected in PGC at protein level, although at 19dpc its expression at RNA level could be barely detected (Fig. 2).

Because DNMT1 was absent in PGC at 14dpc but was detected at 15dpc, we hypothesized that this might represent a moment when PGC DNA is globally remethylated. The de novo DNA methylation depends on the activity of the de novo DNA-methyltransferases DNMT3a and DNMT3b [27, 28]. Thus, we looked at the expression of these enzymes in the rat PGC. As expected, DNMT3a was not detected at 14dpc in protein (Fig. 1d) or RNA level (Fig. 2). Interestingly, DNMT3a labelling was not detected on PGC of in 15dpc embryos at protein level (Fig. 1e) but was detected at the RNA level (Fig. 2). Similarly, this enzyme was detected in 19dpc germ cells, now called gonocytes, at protein level (Fig. 1f), but not at the RNA level, (Fig. 2). DNMT3b immuno-labelling in rat PGC showed the opposite pattern from that observed for DNMT3a, although the pattern of RNA expression was the same for both enzymes (Fig. 2). DNMT3b labelling was detected at 14dpc (Fig. 1g) and at 15dpc (Fig. 1h), although few negative PGC were observed, whereas at 19dpc (Fig. 1i) DNMT3b was not detected in germ cells. DNMT3a and DNMT3b expression at protein level suggests that these enzymes seem to act at distinct phases of rat germ cell development. On the other hand, at RNA level, these enzymes seem to have very similar expression pattern. The inequivalence between protein and RNA data might be a reflexion of technique sensitivity or might be a result of RNA metabolism.

### 5mC and 5hmC Immunolabelling

**Fig. 3:**
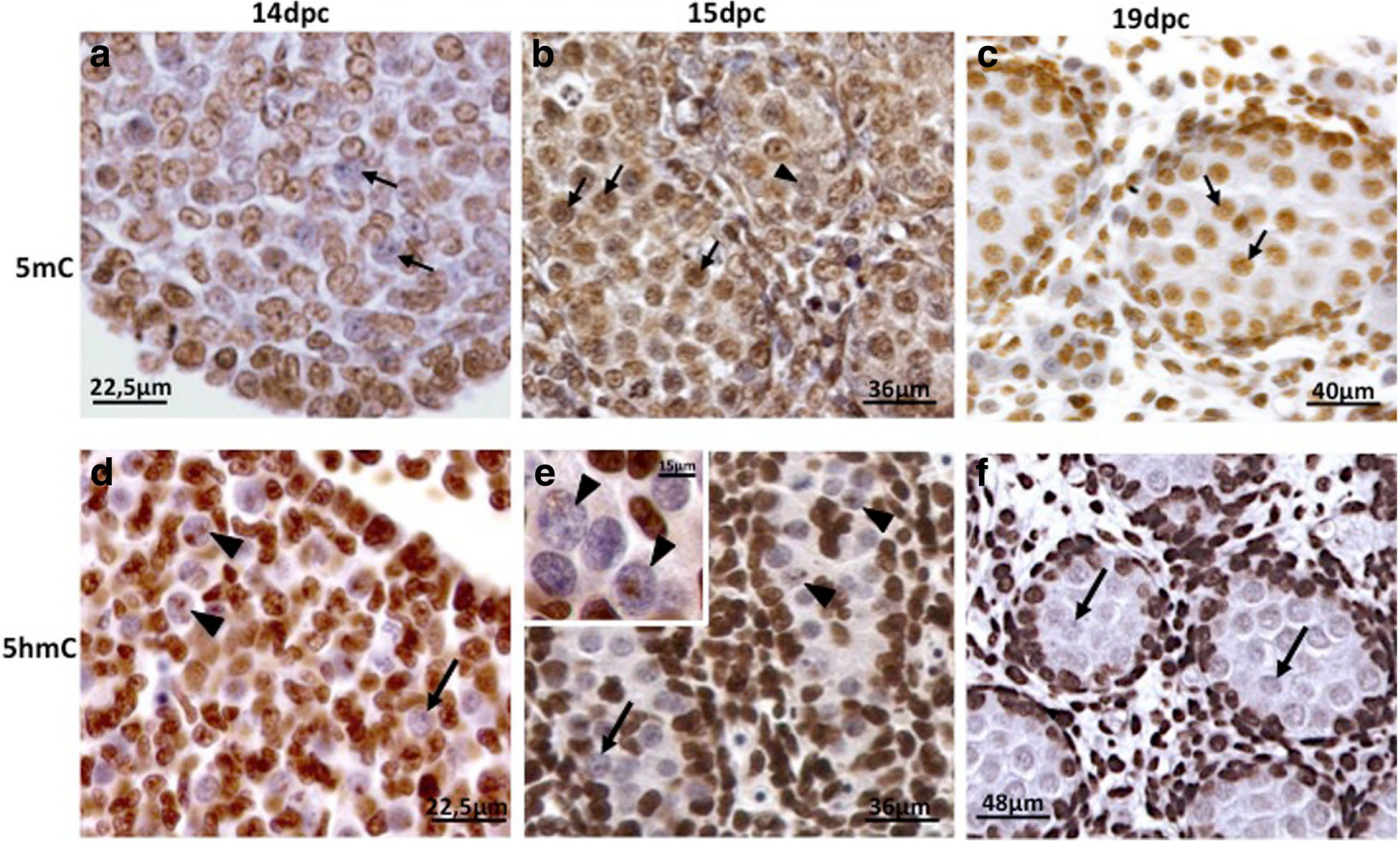
5mC and 5hmC immunolabelling in the embryo gonads at 14dpc, 15dpc and 19dpc. 5mC was not detected in PGC (*arrows*) at 14dpc (**a**). At 15dpc (**b**) 5mC was detected in most PGC (*arrows*), although very few PGC were still negative (*arrowhead*). At 19dpc 5mC was detected in all gonocytes (*arrows*) (**c**). The labelling of 5hmC was restricted to small nuclear areas at 14dpc (**d**) and 15dpc (**e**) PGC (*arrowheads*). 5hmC-negative PGC were also observed (*arrows*). The 5hmC-positive areas were more abundant at 14dpc (**d**) than at 15dpc (**e**). At 19dpc 5hmC was not detected in gonocytes (*arrows*) (**f**)

Because 5-Aza-CdR is expected to promote DNA demethylation through interaction with DNMT1, we investigated 5mC and 5hmC labelling in normal gonads as a basis for the analysis of 5-Aza-CdR effectiveness. 5mC was not detected at 14dpc (Fig. 3a) but appeared at 15dpc (Fig. 3b), when the presence of negative PGC was very rare. At 19dpc all PGC were positive for 5mC (Fig. 3c). These data are somewhat discordant with a recent study showing that rat PGC DNA is hypomethylated from 14.5–19.5dpc [29]. This discrepancy could be due to the different methodological approaches used, such as fixative solutions, which are essential for the detection of this epigenetic mark.

5-hydroxymethylcytosine (5hmC) is the product of 5mC oxidation by TET (Ten-eleven translocation) enzymes. During mouse PGC reprogramming, DNA demethylation depends on 5mC oxidation to 5hmC [16, 17]. In the present study, 5hmC labelling was detected in a restricted area of PGC nucleus from at 14dpc and 15dpc (Figs. 3d and e). The labelling area showed a progressive reduction as the age increased, appearing very rarely at 15dpc until becoming undetectable at 19dpc (Fig. 3f). This agrees with recent data on rat PGC showing that 5hmC labelling is present between 14.5dpc and 16.5dpc but is absent at 19.5dpc [31].

### In Vitro 5-Aza-CdR Administration

The DNMT1 and 5mC data obtained here were used to choose the embryonic age for the in vitro studies using 5-Aza-CdR. Furthermore, we have previously shown that rat PGC migration ends between 15dpc and 16dpc [25]. These findings led us to choose the age of 16dpc for 5-Aza-CdR treatment.

**Fig. 4:**
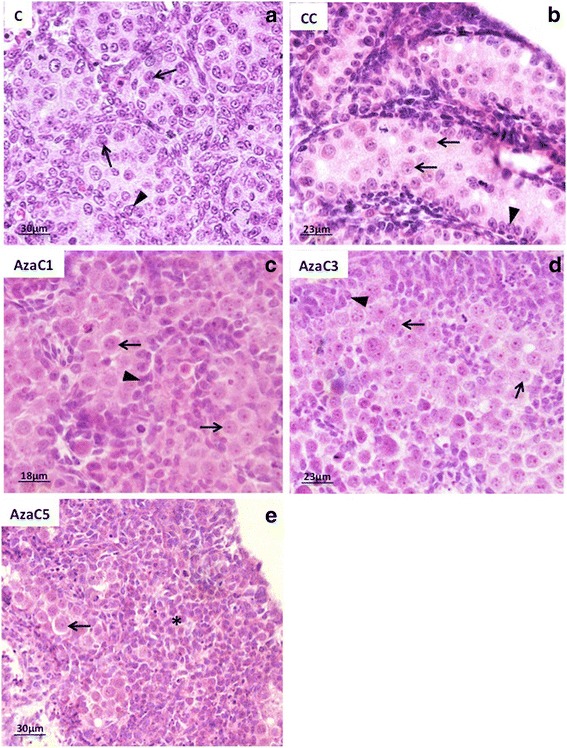
Morphological analysis of the 16dpc gonads of control (C), culture-control (CC) and 5-Aza-CdR-treated gonads. In the C (**a**) and CC (**b**) gonads the seminiferous cords can be clearly distinguished. In the gonads treated with 1 μM fo 5-Aza-CdR (AzaC1; **c**) it still possible to note the cordonal organization, which is less evident in the gonads treated with 3 μM of 5-Aza-CdR (AzaC3; **d**). The gonads treated with 5 μM of 5-Aza-CdR (AzaC5; **e**) very few PGC are observed (*arrow*) and most of the gonad consisted of somatic cells (*)

**Fig. 5:**
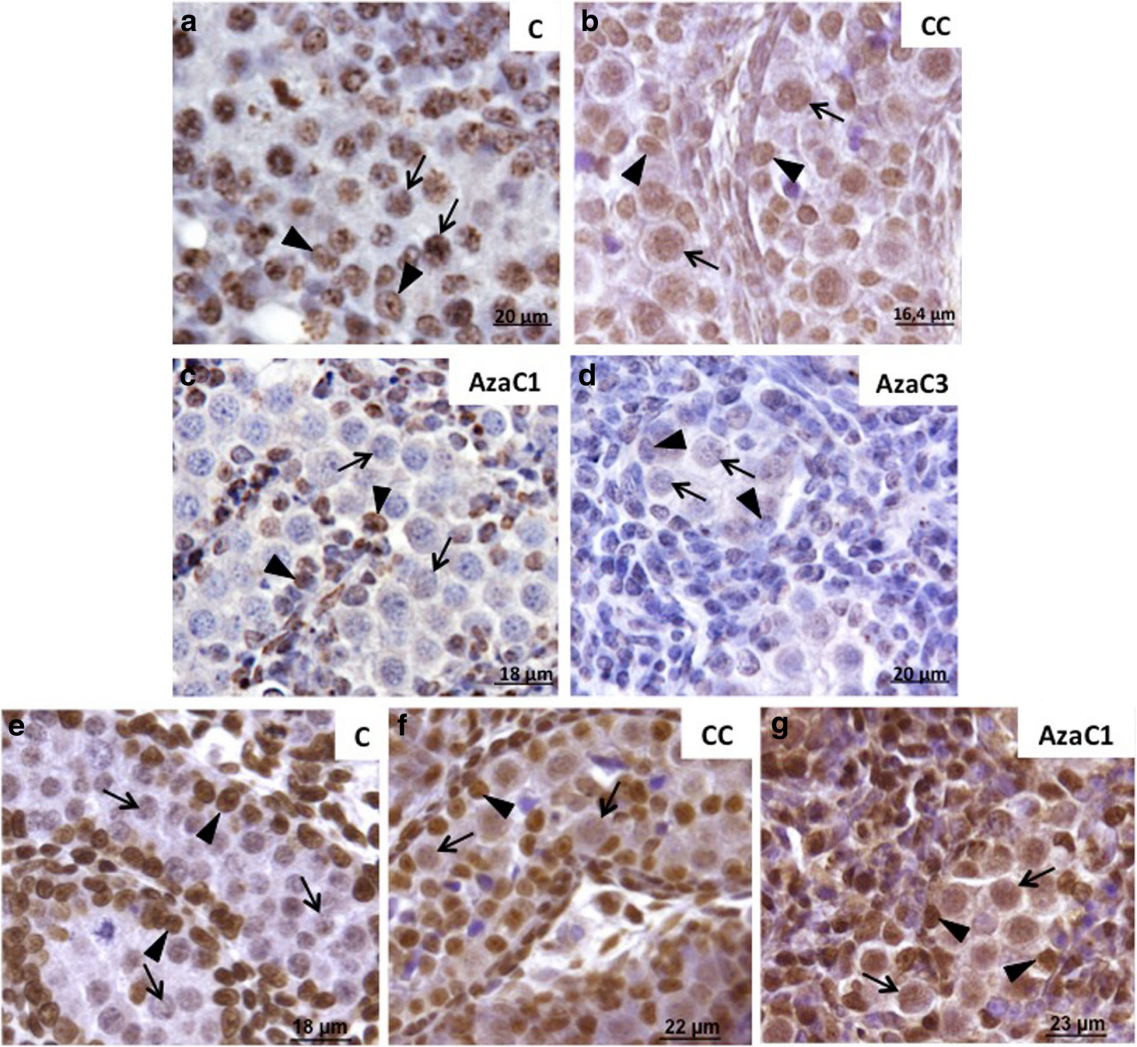
5mC and 5hmC labelling in the control (C), culture-control (CC) and 5-Aza-CdR-treated (Azac1 and AzaC3) gonads of 16dpc embryos. 5mC is detected in C (**a**) and CC (**b**) PGC (*arrows*) and somatic cells (*arrowheads*). In the gonads treated with 1 μM of 5-Aza-CdR (**c**) very weak labelling was observed in PGC (*arrows*) whereas the somatic cells showed more intense labelling (*arrowheads*). The gonads treated with 3 μM of 5-Aza-CdR (**d**) showed weak labelling in PGC (*arrows*) and somatic cells (*arrowheads*). 5hmC was detected in restricted regions of the germ cell nuclei in control (C) gonads (arrows) and in the whole nucleus of Sertoli cells (arrowheads) (**e**). In control-culture gonads (CC) germ cells (arrows) show weak labeling in the whole nucleus, whereas in the Sertoli cells (arrowheads) 5hmC labeling did not change when compared with C gonads (**f**). After 5-Aza-CdR (3μM) treatment (AzaC1), 5hmC labeling was more intense in germ cells (arrows) when compared with the CC gonads, whereas no alteration was observed in Sertoli cells (arrowheads) (**g**)

Morphological analysis of the gonads (Fig. 4) and 5mC labelling (Fig. 5) were used to select the adequate dose of 5-Aza-CdR for the aims of this study. Non-cultured (C) (Fig. 4a) and culture-control (CC) (Fig. 4b) were used as basis for comparison of the 5-Aza-CdR-treated gonads. Three concentrations of 5-Aza-CdR were used (1 μM, 3 μM and 5 μM). The gonads treated with 1 μM (Fig. 4c) showed well-preserved morphology when compared with C (Fig. 4a) and CC (Fig. 4b) gonads. The gonads treated with 3 μM (Fig. 4d) and 5 μM (Fig. 4e) of 5-Aza-CdR showed apparent reduction of its size and morphological alterations, such as cell degeneration (Fig. 4d). In the gonads treated with 5 μM of 5-Aza-CdR PGC were rare and the gonads contained basically somatic cells (Fig. 4e).

### 5mC and 5hmC Labelling after 5-Aza-CdR Treatment

The gonads treated with 1 μM and 3 μM of 5-Aza-CdR were submitted to 5mC immuno-labelling. The gonads treated with 5 μM of 5-Aza-CdR were excluded from this analysis due to the significant germ cell loss, as described above.

As observed for the 19dpc embryos, 5mC was detected in all PGC of the 16dpc embryos (Fig. 5a). After the incubation of embryonic male gonads without 5-Aza-CdR (CC), 5mC was also detected in all PGC and somatic cells (Figs. 5b). On the other hand, 5mC was barely detected in the PGC of the gonads incubated with 1 μM of 5-Aza-CdR (Aza1), but was detected in the somatic cells (Fig. 5c), suggesting that the PGC are more sensitive to 5-Aza-CdR effect on DNA demethylation than the somatic cells. In the gonads treated with 3 μM of 5-Aza-CdR (AzaC3) 5mC labelling was very reduced in both PCG and somatic cells (Fig. 5d), suggesting that this dose is able to induce the loss of 5mC not only in PGC DNA but also in somatic cell DNA. Therefore, we suggest that the dose of 1 μM represents an suitable concentration of 5-Aza-CdR to perform studies involving 5mC detection in rat PGC in whole gonad culture systems.

In control embryo the gonads (C) 5hmC labelling was rarely observed and, when present, was restricted to a small area of PGC nucleus (Fig. 5e). Interestingly, both CC (Fig. 5f) and in AzaC1 (Fig. 5g) gonads showed 5hmC labelling in PGC and Sertoli cells, although it was more intense in Sertoli cells than in PGC. This suggests that the protocol of gonad culture used here leads to an increase of 5hmC detection in PGC.

### PGC Quantification and Proliferation

**Fig. 6:**
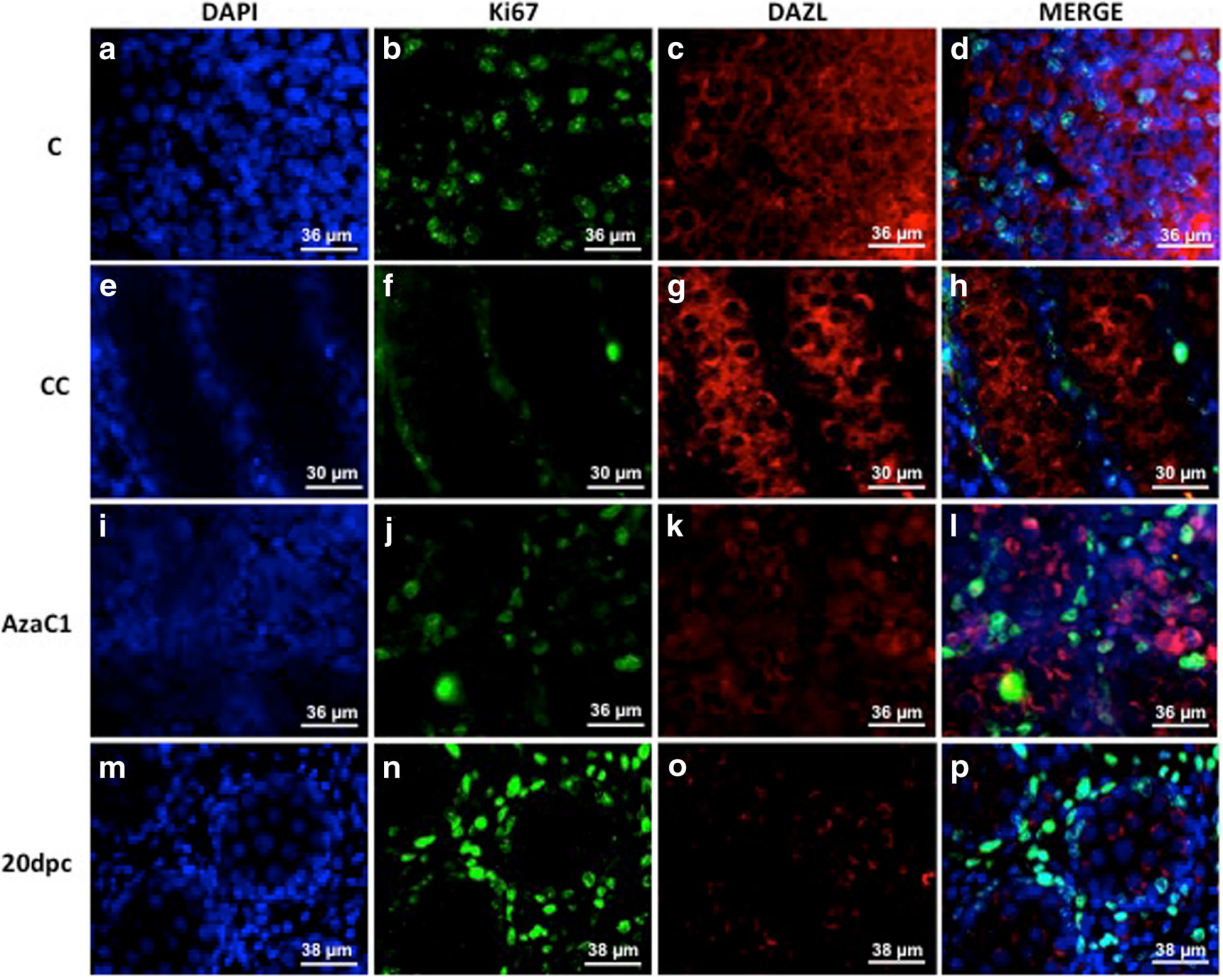
Ki67/DAZL immunolabelling the control (C) (**a** to **d**), culture-control (CC) (**e** to **h**), 5-Aza-CdR-treated gonads (AzaC1) (**i** to **l**) and 20dpc (**m** to **p**). Ki67 was detected in DAZL-positive cells in C gonads but not in CC, AzaC1 and 20dpc gonads. (**a**), (**e**), (**i**) and (**m**): DAPI; (**b**), (**f**), (**j**) and (**n**): Ki67 labelling; (**c**), (**g**), (**k**) and (**o**): DAZL labelling; (**d**), (**h**), (**l**) and (**p**): merge

**Fig. 7:**
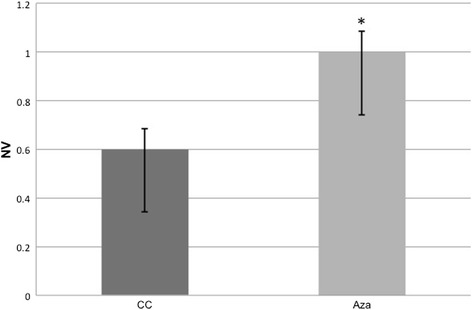
PGC quantification in 16dpc culture-control (CC) and Aza-treated (Aza) embryo gonads. An increase in the numerical density (Nv) of PGC is observed in the Aza-treated gonads

Because the morphological analysis of the AzaC1 gonads suggested an increase of PGC number, we decided to count these cells using a germ cell marker (DAZL) associated with a proliferation marker (Ki67). The Ki67/DAZL double-labelling indicated that PGC proliferation was active in the C gonads (Figs. 6a to d), but was inactive in both CC (Figs. 6e to h) and AzaC1 gonads (Figs. 6i to l). If germ cells continue to develop in vitro, it is indeed expected that their proliferation has ceased by the end of the culture period, when these cells would correspond to 20dpc germ cells. At this age rat male germ cells has entered mitotic arrest (Zogbi et al. [30]). The Ki67/Dazl double-labelling was then performed in 20dpc embryos to confirm this data. Indeed, no Ki67 labelling was observed in the 20dpc germ cells (Figs. 6m to p). Despite the fact that PGC were Ki67-negative by the end of the culture period, the number of PGC in the AzaC1 gonads was higher when compared with the control gonads (Table 1), what lead to an increase of the numerical density (Nv) of these cells in the AzaC1 gonads (Fig. 7). This suggests that 5-Aza-CdR caused an alteration of germ cell proliferation, although their maturation, which can be indicated by mitotic arrest, which was not impaired.

**Table 1:** Area and primordial germ cell count (N) averages in CC and AzaC1 groups

	CC	AzaC1
Area	238,9 ± 7,0	256,8 ± 6,7
N(PGC)	149,8 ± 7,4	273,5 ± 16*

The gonad area was not different between CC and AzaC1 groups. However, the number of PGC was higher in AzaC1 gonads compared with CC gonads

*p<0.05

### Expression of Developmental Genes

**Fig. 8:**
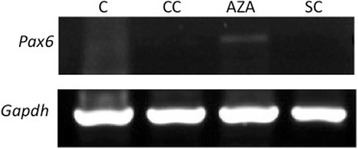
Expression of the *Pax6* developmental gene in the PGC isolated from control (C), culture-control (CC) and Aza-treated (Aza) gonads. Pax6 expression was detected in Aza PGC but was absent in C and CC gonads. Somatic cells (SC) obtained from Aza gonads gonads were used to check the PGC-specific activation of *Pax6* by 5-Aza-CdR

The expression of developmental genes is a mark of the early phases of embryo development and of embryonic stem cells. In mouse PGC these genes are not expressed, although seems to be maintained in a bivalent state [31, 32]. Since DNMTs are fundamental for the control of developmental genes expression during embryo development [33], we investigated whether 5-Aza-CdR treatment induces the expression of the somatic developmental gene Pax6 in rat PGC. *Pax6* expression was not detected in the control (C and CC gonads) PGC or in the somatic cells. On the other hand, in the in the AzaC1 gonads *Pax6* expression was detected in PGC but not in the somatic cells (Fig. 8), suggesting that the treatment with 5-Aza-CdR induced PGC-specific activation of this gene.

## Discussion

Germ cell development depends on strictly controlled events that assure their normal differentiation and consequently the production of functional gametes and healthy embryos. In this context PGC epigenetic reprogramming is of particular importance, since it is required for PGC sex differentiation, imprinting establishment and to provide adequate chromatin conditions for the future embryo development. Many important studies have been investigating and describing PGC epigenetic reprogramming, especially in the mouse, and have provided substantial data. However, studies about rat PGC epigenetics are very scarce, probably due to the reduced information about molecular aspects of the rat development when compared to mouse. This lead us to search for methodologies to study rat PGC epigenetics. Here we used 5-Aza-CdR, which is known to interact with DNMT1 and to block its activity, to promote male PGC DNA demethylation in vitro. To propose the protocol used here, we took into account two main aspects: the difficulties of getting enough number of PGC during early reprogramming and the fact that DNA methylation represents a major form of gene expression control.

PGC reprogramming seems to involve the reduction of the activity of DNMT1 in the beginning of the reprogramming period [13, 34] followed by the recruiting of DNMT3a and DNMT3b [18, 35, 36]. Since 5-Aza-CdR inhibits DNMT1 activity and also affects DNMT3a and DNMT3b [37, 38], it was necessary to investigate the expression of these enzymes prior to establish the protocol of 5-Aza-CdR treatment in the rat gonad cultures. We detected DNMT1 in rat PGC at 15dpc and 19dpc, but not at 14dpc. Since DNMT1 is described as the maintenance DNMT, it is expected that its presence correlates with the moment of PGC de novo DNA methylation. However, a previous study showing epigenetic marks in rat PGC indicated that 5mC reestablishment occurs around 19.5dpc [39], suggesting DNA de novo methylation occurs in late gestational period. On the other hand, in mouse PGC, DNMT1 was detected between 12.5dpc and 16.5dpc [19], when DNA is globally demethylated [13], what agrees with our data. Conversely, [18] suggested that DNMT1 is not present in mouse PGC between 14.5dpc and 18.5dpc, a period that includes demethylated and de novo methylated DNA [13]. DNMT1 expression agrees with 5mC labelling, which was not detected at 14dpc and was detected at 15dpc and 19dpc. These data suggest that rat PGC are hypomethylated at 14dpc and that de novo methylation may start between 14dpc and 15dpc.

The de novo methyltransferases DNMT3a and DNMT3b are fundamental for de novo methylation of the mouse embryo [29] and ES cells [40] although seems to be dispensable for somatic cell nuclear reprogramming to a pluripotent state [41]. In the mouse germ cells DNMT3a, DNMT3b and DNMT3l are important for de novo methylation of imprinted regions during development and after birth and for retrotransposons silencing [42]. DNMT3a was reported to act in the acquisition of de novo methylation in mouse PGC [18, 20], whereas DNMT3b seems to function in the maintenance of methylation [20] and to play a major role in the postnatal development of male germ cells [18]. Our data on DNMT3a expression suggest that this enzyme is not present in PGC at 14dpc, what agrees with the 5mC data. However, at 15dpc and 19dpc the expression of this enzyme at both protein and RNA level is controversial, indicating that further studies are needed. In contrast DNMT3b was detected at 14dpc and 15dpc but not at 20dpc, showing an opposite pattern to that observed for DNMT3a. This suggests that these de novo DNA-methyltransferases seem to play distinct roles in rat germ cell development.

PGC reprogramming in mice seems to be associated with changes in the expression of the DNMTs and their partners. The downregulation of *Uhrf1*, which is essential for DNMT1 activity, was observed in mice PGC in the beginning of epigenetic reprogramming [43], suggesting that DNMT1 action needs to be annulled at this stage of germ cell development. Thus, the use of a DNMT1 inhibitor seems to be a reasonable way to mimic, at least in part, the global DNA demethylation that occurs during PGC reprogramming. Our analyses suggest that the incubation of rat embryonic gonads with the DNMT1 inhibitor 5-Aza-CdR was able to induce wide loss of 5mC in PGC DNA, without causing major global loss of this mark in somatic cells, as suggested by 5mC immno-labelling. The detection of *Pax6* expression, which is controlled by promoter methylation [44], observed in PGC but not in the somatic cells of the gonads treated with 5-Aza-CdR on in the control PGC corroborate to the hypothesis that 5-Aza-CdR treatment acted primarily on PGC. This suggests that in vitro treatment of whole-gonad with 5-Aza-CdR might be an interesting method to study the control of gene expression by DNA methylation in PGC.

Considering that 5-Aza-CdR is an inhibitor of DNMT1 activity, further studies investigating the pluripotency of PGC after 5-Aza-CdR treatment seems to be another point to be considered, since it has been shown that the absence of DNMTs leads to spontaneous pluripotency [45].

On the other hand, the alteration of 5hmC labelling even in the control culture gonads indicates that the culture method by itself is able to induce epigenetic changes in PGC. This and other aspects of PGC epigenetics, such as histone mark profile, need to be further investigated to confirm whether this model would indeed be useful to study PGC epigenetics.

## Conclusion

In conclusion, we describe the immuno-labelling of DNMTs in rat PGC and suggest that the administration of 5-Aza-CdR to rat gonads in vitro leads to a wide demethylation of PGC DNA without major effects on somatic cells. We finally suggest that this method might be a potential alternative method to study DNA methylation and demethylation, although additional studies are necessary to validate it.

## Methods

### Animals and Tissue Preparation

Male embryos were obtained from timed matings of adult Wistar rats (*Rattus norvegicus albinus*) from the Laboratory of Developmental Biology (EPM/UNIFESP, Sao Paulo – Brazil). The adult animals were kept in plastic cages under a 12–12 h light/dark cycle at 23–25 °C. Food and water were allowed ad libitum. The dams were anesthetized using the method of anaesthesia/analgesia (xylazin/ketamine, 10 mg/Kg and 100 mg/Kg, respectively) and euthanized by cardiac incision. The embryos were collected at 14, 15, 16 and 19dpc. At 14dpc the rat embryos do not show sexual dimorphism. At 15, 16 and 19dpc sexing was performed by morphological inspection of the gonads and only male gonads were used. The gonads of 14, 15 and 19dpc embryos were fixed in Bouin’s solution for 6 h or in Carnoy’s solution for 48 h and processed for paraffin embedding. Cross sections (6 μm-thick) were obtained from embryos and testes and submitted to the labelling of DNMT1, DNMT3a and DNMT3b (see item 4.3). Five embryos from three different mothers were used for this analysis.

The male gonads of 16dpc embryos (20 embryos from 6 different mothers) were dissected and incubated with 5-Aza-2′-deoxicytidine (5-Aza-CdR) as described below (see item 4.2). The experiments were carried out under the rules of the local committee for animal care (CEUA Nr. 7,001,040,914).

### 5-Aza-CdR Experiment

The uterus of 18 pregnant females at gestation day 16 (GD16) were removed and taken to a culture room pre-sterilised with UV light. The gonad cultures were performed according to Livera et al. [26] and Habert et al. [27]. The experiment was performed three times, using 6 dams for each experiment (total of 18 dams). A total of 72 male embryo gonads (24 gonads per experiment) were dissected and placed on gridded cellulose/ester membrane (0.45 μm, HAWG047S0, Millipore, USA) in 24-well plates containing DMEM supplemented with GIBCO® GlutaMax (Life technologies), 0.5% BSA, 1% Penicillin/Streptomycin and 1 μM, 3 μM or 5 μM of 5-Aza-CdR (InSolution™, Cat. 189,826 - Calbiochem). These gonads are from here on referred as AzaC. Control culture (CC) gonads were performed using the same culture medium except for the addition of 5-Aza-CdR. The cultures were maintained for 5 days. 5-Aza-CdR was added on the 1st day of culture and replaced every day. The control and 5-Aza-CdR-treated gonad cultures were carried out concomitantly in every experiment, which was performed three times.

Six control and six 5-Aza-CdR-treated gonads (two from each experiment) were fixed in Carnoy’s solution and embedded in paraffin for 5mC and 5hmC labelling and Haematoxilyn and Eosin (H&E) staining. Other 30 control and 30 5-Aza-CdR-treated gonads (10 from each experiment) were used for PGC purification and RT-PCR (see item 4.4). The gonads were dissected from 20 male embryos from 6 different mothers.

### Immunohistochemistry and Immunofluorescence

The sections were dewaxed in xylene, hydrated and submitted to heat antigen retrieval using citrate buffer (pH 6.0) for 10 min (for DNMT1, DNMT3a, DNMT3b and H3K27me3) or to proteinase K (20 μg/ml) for 5 min (for 5mC and 5hmC). Antigen blocking was performed using 5% BSA and the slides were incubated with primary antibodies: anti-5mC (1:50, Abcam, ab73938), anti-5hmC (1:50, Active Motif, 39,769), anti-DNMT1 (1:100, Santa Cruz, sc-20,701), anti-DNMT3a (1:100, Santa Cruz sc-20,703) anti-DNMT3b (1:100, Santa Cruz, sc-20,704), anti-Ki67 (Abcam, ab16667) and anti-DAZL (1:200, Serotec, MCA2336) overnight at 4 °C. For immunohistochemistry the slides were washed in PBS (0.05 M, pH 7.2) and incubated with the LSAB system (DAKO Detection System - K0690) and then with streptavidin-peroxidase (LSAB, DAKO) for 30 min. The reactions were revealed with DAB (DAKO). For immunofluorescence, after two PBS washes, the slides were incubated with the secondary antibodies Alexa (Invitrogen, A10036) anti-mouse and FITC (Abcam, Ab6791) anti-rabbit. The nuclei were stained with DAPI.

Because of protocol and antibody incompatibilities the double labelling of the methylation marks and PGC markers was not possible. Thus, germ cells were identified by their typical morphology (round nucleus and prominent nucleolus) and localization.

The slides were carefully analysed and the pattern of protein detection in germ cells was described using the Image Analysis System Leica QWin V3 (Cambridge, England) for immunohistochemistry and the NIS Element (Nikon) for immunofluorescence.

### PGC Quantification and Proliferation

The Nv represents the number of cells in a given volume of tissue. PGC in the CC and AzaC gonads were counted and a numerical density (Nv) of these cells was obtained by the ratio between the number of PGC and the volume of the gonad tissue analysed (Zogbi et al. [46]). The quantification was performed in the Ki67/DAZL double-stained gonads.

### Two-Step PGC Sorting and RT-PCR

After 5 days in culture, the gonads were removed from the membrane dissociated in collagenase/dispase solution (10,269,638,001, Roche) at 37 °C, for 20 min. The cell suspension was incubated for 2 h in 12-well plates pre-treated with gelatin (G1890, Sigma) for somatic cell adhesion. The medium containing non-adhered cells (including PGC) was centrifuged and resuspended in 1X PBS (pH 7.2) containing magnetic beads pre-incubated with biotinilated *Dolichus biflorus* agglutinin (DBA). The suspension was incubated for 1 h at 4 °C rocking and placed in a magnetic rack (Invitrogen). The positive selected cells (PGC) and the negative selected cells (somatic cells) were submitted to RNA isolation using TRIzol® (Invitrogen). The cells that adhered to the 12-well plates in the first step of the selection were treated with 1X trypsin (59418C, Sigma) and combined with the magnetic negative selection cells for RNA isolation. The cDNA was obtained using the Superscript III Reverse Transcriptase (Invitrogen). Conventional PCR was performed using specific primers for the developmental genes Pax6 (FP 5′-agtgaatgggcggagttatg-3′ and RP 5′-aacaaccacatgagccaaca-3′) and Dazl (FP 5′-gaaatggcccacaaaagaaa-3′ and 5′-ttaagcactgcccgacttct −3′).
